# Genome-wide analysis of FATA associated with drought tolerance in tetraploid potato (*Solanum tuberosum*)

**DOI:** 10.3389/fpls.2026.1917461

**Published:** 2026-08-25

**Authors:** Florian Schilling, Annika Reimer, Renate Horn

**Affiliations:** Institute of Biological Sciences, Department of Plant Genetics, University of Rostock, Rostock, Germany

**Keywords:** cuticle, drought tolerance, FATA, haplotypes, potato, SNP

## Abstract

The cuticle represents the outer most protective barrier against biotic and abiotic stresses. It is composed of cutin and waxes and protects plants from desiccation, UV, cold, mechanical stresses, and pathogens. GWAS/BSAseq combined with SeqSNP analyses in an association panel of 34 potato cultivars had revealed that the acyl-ACP thioesterase FATA (Soltu.DM.06G033680.1) is significantly associated with drought tolerance in potato. Apart from three *FATB* genes, only one *FATA* gene is present in potato that has the highest homology to *FATA2* in Arabidopsis. FATA is responsible for the export of C18:1 fatty acid from chloroplast into cytosol, which is necessary for the biosynthesis of cutin. A knockout mutant of *AtFATA2* was analyzed with regard to the cuticle permeability and to drought tolerance as well as recovery. Loss of FATA function leads to higher sensibility to water deficit in Arabidopsis, but to no change in recovery. The increased permeability of the cuticle in the *fata2* knockout mutant as shown indirectly by higher chlorophyll leaching might play a role in this. Haplotypes for FATA were identified for the two potato cultivars Albatros and Désirée. All Désirée haplotypes and Albatros haplotypes 1, 3 and 4 were also revealed by former potato pan genome studies, while Albatros haplotype 2 is unique and has not been described before. Protein models were developed to investigate the influence of different SNPs in the haplotypes on the predicted protein structure and especially the substrate cavity. In potato, protein modeling suggests that only the hypothetical isoform B of FATA might be able to process oleoyl-ACP, but not hypothetical isoform A. However, this hypothesis needs to be verified by enzyme activity assays.

## 1 Introduction

Potato (*Solanum tuberosum*) is the sixth most produced crop in the world and plays a major role in feeding the world population (Faostat, 2023). In recent years, the demand for potato has increased in developing countries in Africa, Asia and South America, because potato tubers represent a valuable resource for starch, fibre, minerals, proteins, vitamins and secondary plant metabolites (Beals, 2018). However, climate change with longer lasting drought periods makes cultivation of potatoes more difficult and threatens potato yields around the world. Due to a shallow root system potato plants suffer severely under water deficits (Zarzyńska et al., 2017). Increasing frequency and length of drought periods have a grave impact on the growth of potato plants, the tuber yield and its quality (Wang et al., 2024b). With a water content of 80% in the tubers the need for water is high (Gopal and Iwama, 2007; Watkinson et al., 2008). Therefore, in some locations irrigation systems are used to compensate the lack of water, which represents only a short-term solution due to decreasing groundwater levels and problems with soil salinization (Billiau et al., 2012; Sun et al., 2022).

Development of potato varieties that are more tolerant to drought stress will therefore have a decisive influence on the food security of the world population in the coming years (Ibrahim Ibrahim et al., 2023). More knowledge is required about the genes responsible for resistance to drought to drive this process. This is the only possibility to respond in a timely manner to the accelerated climate change and its consequences. Molecular markers for a number of genes involved in drought response in potato have already been developed (Islam et al., 2024). Utilizing SNP based markers allowed the identification of seven drought stress related QTLs (Anithakumari et al., 2011). Association studies in a panel of 34 German starch potato cultivars identified five candidate genes *ACS3*, *ALDH*, *ETRTF3*, *PARG* and *PP2C* for drought tolerance in potato (Schumacher et al., 2021). The roles of ACS3 and PP2C were further studied in mutants using virus-induced gene silencing and confirmed their interaction and importance in drought stress in potato (Hamera et al., 2025). Additional 11 candidate genes for drought tolerance in potato could be revealed by applying BSAseq for QTL analyses and selected candidate genes could be additionally verified by SeqSNP analyses in an association panel of 34 potato cultivars (Schilling et al., 2024). Most of these genes (*StABP1*, *StKS, StLEA, StPKSP1*, *StPKSP2*, *StBRI1, StYAB5*, and *StZOG1*) are involved in plant development, but three of the genes (*StFATA, StSYP* and *StHGD*) seem to be responsible for plant protection as e.g. Soltu.DM.06G033680.1 encoding the acyl-ACP thioesterase FATA. In plants, the *de novo* fatty acid synthesis takes place in the stroma of chloroplasts. The fatty acid synthase complex elongates and esterifies acyl intermediates to acyl carrier proteins (ACP) (Ohlrogge and Jaworski, 1997). However, glycerolipids and very long fatty acid chains (VLCFA) are synthesized in the endoplasmatic reticulum, so the synthesized fatty acids need to be exported from the plastids to the cytosol. This process requires acyl-ACP thioesterases, which hydrolyse acyl-ACPs and release free fatty acids and reduced ACPs (Somerville and Browse, 1991; Zhou et al., 2024). There are two gene families of acyl-ACP thioesterases, FATA and FATB, both are encoded by nuclear genes and imported into plastids (Jones et al., 1995; Sagun et al., 2023; Xu et al., 2023). FATA thioesterases can be found throughout the plant kingdom, with a high substrate specificity for oleoyl-ACP that makes them responsible for the export of C18:1 fatty acids from the chloroplasts (Hellyer et al., 1992; Salas and Ohlrogge, 2002; Sanchez-Garcia et al., 2010). On the other hand, FATB thioesterases have a higher variety in substrates and have been more intensely studied (Li-Beisson et al., 2013; Ma et al., 2021; Tang et al., 2022) than FATA, which has been mostly neglected. FATB thioesterases can process both palmitoyl-ACP and oleoyl-ACP (Jones et al., 1995; Dormann et al., 2000). Genetic engineering was successfully applied to change the substrate specificity in acyl-ACP thioesterases (Yuan et al., 1995; Thelen and Ohlrogge, 2002; Feng et al., 2017; Jing et al., 2018).

The cuticle represents the outer most protective barrier of the plant against biotic and abiotic stress (Arya et al., 2021; Zhao et al., 2025). The two main components of the cuticle are cutin and waxes, which are deposited on the outer cell wall of epidermal cells (Yeats and Rose, 2013). The cuticular wax is composed of two layers, a top layer of crystallized epicuticular wax and the intracuticular wax, which is embedded within the cutin scaffolds (Jetter and Schäffer, 2001). Saturated C16 and C18 fatty acids are utilized in the fatty acid elongation to produce VLCFA, which are needed for the synthesis of aldehydes, alkanes, primary and secondary alcohols, wax-esters and ketones, which are then used to form cuticular waxes (Wang et al., 2012; Li-Beisson et al., 2013). Unsaturated C18:1 and saturated C16 fatty acids are used to produce sn-2 mono(oxygenated)acyl-glycerol, which represents the main monomer in cutin polymers (Yeats et al., 2014). FATA has a major impact on providing these fatty acids for the cutin matrix, but only marginally participates in the synthesis of intra- and epicuticular waxes (Li-Beisson et al., 2013). The strong hydrophobic properties of the cuticle protect plants from desiccation (Joubès and Domergue, 2018). Cutin polymers offer a mechanically stable matrix, which is connected to the cell wall and allows the embedding of waxes. The embedded waxes primarily protect the plant from water loss by non-stomatal transpiration (Schonherr, 1976; Dominguez et al., 2015). In addition, the epicuticle waxes protect plants from pathogens and UV (Dominguez et al., 2017; Domanda et al., 2024).

*A. thaliana* plants exposed to drought stress accumulated approximately 75% more total leaf cuticle wax than nontreated plants. Up to 98% of the increased amount of waxes consist of alkanes. Apart from waxes, leaf cutin monomers increased in drought stressed *A. thaliana* plants by 65% (Kosma et al., 2009). Leaf waxes composition and accumulation rates in *Brassica napus* changed dramatically under combined drought and heat stress, with C16 fatty acids being increased under drought stress (Tomasi et al., 2024). Comparisons of cotton varieties showed increased accumulation in leaf waxes under water deficit. The cutin monomers palmitic acid and stearic acid were increased under drought stress, while linolenic acid and linoleic acid were repressed (Yang et al., 2022). Differential expression analyses in *Triticum aestivum* revealed that fatty acyl-CoA reductase (FAR)-like genes involved in the synthesis of wax esters are increased under drought and cold stress (Tian et al., 2024). Comparisons of glaucous and non-glaucous wheat NILs showed up to 151.1% increased cuticular waxes under drought stress. It was also revealed that plants with higher amounts of accumulated cuticular waxes under drought stress were able to maintain PSII activity for a longer time (Guo et al., 2016). Whereas Bi et al. (2017) suggests that specific wax compositions are most important, Zhao et al. (2025) addresses the sheer amount of cuticular waxes. In Arabidopsis, knockout mutants of genes involved in cuticle biosynthesis like *ALDH2C4* and *DREB26SR* made the plants more susceptible to drought, whereas the drought tolerance increased when genes of cuticle synthesis were overexpressed (Khan et al., 2026). A drought resistant *Nicotiana tabacum* EMS mutant showed less stomatal conductance and transpiration rate while maintaining a higher photosynthetic rate (Hu et al., 2022). The overexpression of *NtCaM13* in *N. tabacum* lines resulted in a significant upregulation of the long-chain alcohol oxidase FAO4A, which is involved in the cutin synthesis (Li et al., 2025). Another study by Khan et al. (2020) showed that drought-hardening improved the overall drought tolerance of *N. tabacum* plants and was accompanied by changes in the leaf anatomy. Genome edited *Nicotiana glauca* mutants of the wax metabolism were also investigated with regard to their cutin composition as a number of precursors and enzymes are shared between the wax and the cutin metabolism (Negin et al., 2023). The mutant *cer1* showed significant differences in the composition of cutin monomers. The mutant demonstrated threefold increased rate in water loss (Negin et al., 2023).

In *Solanum lycopersicum*, transcriptome analysis demonstrated that FATA located in the plastids is upregulated by a factor of 1.7 under drought conditions (Asakura et al., 2021). This was accompanied by an increased expression of other genes of the fatty acid metabolism, namely enoyl-ACP reductase (*FabI*), 3-oxoacyl-ACP synthase I (*FabB*) and acyl-ACP-desaturase (*AAD*). *FATB1* and *FATB3* knockout tomato mutants showed changes in the epicuticular wax structure, with a decline of protective waxes. This was accompanied by reduced density of trichomes and stomata (Asakura et al., 2021; Bahadir et al., 2024). In *Zanthoxylum bungeanum*, acyl-ACP thioesterases are involved in the cold response by changing the cell membrane composition and thus maintaining membrane fluidity (Tian et al., 2022).

However, the role of acyl-ACP thioesterases has been mostly investigated with regard to seeds. In *A. thaliana*, two gene copies of *FATA* (AT3G25110 and AT4G13050) were identified and one copy of *FATB* (AT1G08510.1) (Beisson et al., 2003). Arabidopsis *FATA* double mutants have shown different amounts of fatty acids and compositions in seed oil compared to the wild type (Moreno-Perez et al., 2012). In *Paeonia lactiflora* ‘Hangshao’, transcriptome analyses revealed also the presence of two differentially expressed genes (DEG) for FATB, but only one for FATA during seed development (Meng et al., 2021). The highest expression was observed 45 days after flowering. *FATA* is regarded by the authors as one of the key genes for synthesis of unsaturated fatty acids and oil content in *Paeonia* seeds. Investigating the fatty acid biosynthesis in *Perilla frutescens* var. *frutescens*, an Asiatic oil crop belonging to the mint family, transcriptomic analyses, revealed two expressed genes for fatty acyl-ACP thioesterases, *FATA* (Locus_29919) and *FATB* (Locus_6603), which correspond to *AT3G25110.1* (*FATA1*) and *AT1G08510.1* (*FATB*), respectively (Bae et al., 2022). Recently, two acyl-ACP thioesterases, GmFATA1 and GmFATA2, were characterized in soybean (*Glycine max*) (Liao et al., 2024). The two homoeologous enzymes showed overlapping, but only partial functional redundancy. Gene editing mutations of either GmFATA1 or 2 led to early growth defects in the soybean seedlings, whereas overexpression of GmFATA significantly increased vegetative growth, seed fatty acid content and seed yield (Liao et al., 2024).

In potato, little is so far known about the role of fatty acyl ACP thioesterase in the formation of the cuticle and the role under drought conditions. In this study, we characterize acyl-ACP thioesterases in *S. tuberosum* with special emphasis on the drought-stress associated *StFATA* and its homologues in *A. thaliana*, *S. lycopersicum, N. tabacum, Nicotiana benthamiana* and *T. aestivum*. We investigated the role of *AtFATA2*, the closest homolog to *StFATA*, in an Arabidopsis knockout mutant under drought stress and recovery conditions. *N. tabacum*, *N. benthamiana* and *T. aestivum* have been added due to the well-established drought stress research in these species (Zhang et al., 2011; Liu et al., 2024; Yin et al., 2025; Wang et al., 2026). Due to the tetraploid genome of *S. tuberosum*, SNP-based haplotypes of *StFATA* were derived for Albatros and Désirée. For the two hypothetical isoforms A and B different substrate specificities were postulated. The dosage effect of the presence of isoform A on drought tolerance was investigated. The results indicate that it might be very interesting to further study the role of FATA for the formation of the cuticle in potato and its possible role in drought tolerance.

## 2 Materials and methods

### 2.1 Plant material

Potato cultivars Albatros and Désirée were obtained from Norika, Sanitz, Germany and grown in the greenhouse (16 h light/8 h dark; 22 °C at day time, 18 °C at night time). Tuber skins and leaves were harvested for DNA and RNA-extractions and shock frozen in liquid nitrogen. The material was stored at -80 °C until further use.

For the characterization of *FATA2*, the *A. thaliana* T-DNA mutant SALK_111046C as well as the wild type (Colombia-0) were ordered at the Nottingham Arabidopsis Stock Centre (NASC). After stratification of the seeds for 2 days in the dark at 5 °C, plants were grown in a climate chamber under long day conditions with 16 hours daylight/8 hours night cycle at a light intensity of 100–150 µmol·m^-2^·s^-1^ at 20 °C/22 °C for several weeks.

### 2.2 Comparison of acyl-ACP thioesterase genes in potato, Arabidopsis, tomato, tobacco, *Nicotiana benthamiana*, and wheat

Information about acyl-ACP thioesterase genes was gathered in three different ways. Starting point was the potato candidate gene (Soltu.DM.06G033680.1) associated with drought tolerance in Schilling et al. (2024). First, sequences were retrieved from SpudDB (Hamilton et al., 2025) and mapped with the candidate gene sequence against the reference sequences of potato (Pham et al., 2020), Arabidopsis (Rhee et al., 2003), tomato (The Tomato Genome Consortium, 2012), tobacco (Wang et al., 2024a), *Nicotiana benthamiana* (Wang et al., 2024a) and *Triticum aestivum* (The International Wheat Genome Sequencing Consortium, 2018) using blastp 2.16.0 (Altschul et al., 1990). The next step was a domain search using HMMER 3.4 again using the reference sequences of potato, Arabidopsis, tomato and wheat with the PLN02370 superfamily (Eddy, 2011) to identify additional homologues. The last step was a semantic search using the Ensemble database (Dyer et al., 2024). Further information like e.g. the isoelectric point (pI), protein sequence length and molecular weight (MW) were calculated using the ProtParam tool under the ExPASy web server (Duvaud et al., 2021). Transfer peptides were identified for *StFATA, StFATB2* and *StFATB3* using TargetP – 2.0 (https://services.healthtech.dtu.dk/services/TargetP-2.0/).

### 2.3 Phylogenetic studies, gene structures, motifs, chloroplast transit peptide prediction

For the construction of the phylogenetic tree, protein sequences of *S. tuberosum*, *S. lycopersicum*, *A. thaliana, N. tabacum*, *N. benthamiana* and *T. aestivum* were compared. For the alignment of all sequences MEGA11s’ Multiple Sequence Comparison by Log-Expectation (MUSCLE) algorithm was used (Tamura et al., 2021). In addition, construction of the phylogenetic tree was performed with the Maximum Likelihood method with a bootstrap value of 1000.

### 2.4 Cis-active element identification

Promoter sequences from the doubled monoploid potato *S. tuberosum* Group Phureja DM 1-3–516 R44 were analysed in PLACE (Higo et al., 1999). From phytozome, 2000 bp upstream of each start codon from *StFATA*, *StFATB1*, *StFATB2*, *StFATB3* of DM 1-3–516 R44 v6.1 (Pham et al., 2020) and from *StFATA* of the cultivars Albatros and Désirée (SRR14400529 and SRR37799278) were extracted and analysed for conserved motifs (Goodstein et al., 2012).

### 2.5 Differential expression analysis

Expression data for *StFATA*, *StFATB1*, *StFATB2* and *StFATB3* of the potato cultivar Désirée were extracted from Sprenger et al. (2016) and of the potato cultivar Lady Rosetta from Aliche et al. (2021). Means, log_2_FoldChange (log_2_FC) and p-values between drought stressed and control plants were calculated for Désirée with the given FPKM-values for three biological replications in the separate field and greenhouse trials. For the study of Lady Rosetta, means, log_2_FC and p-values between drought stressed and control plants were taken from the **Supplementary Data Sheet** (Aliche et al., 2021).

### 2.6 Characterization of *FATA* from potato

DNA and RNA were extracted from potato tuber skins and leaves of Albatros and Désirée. RNA extraction followed the protocol from Kumar et al (Kumar et al., 2007). Synthesis of cDNA was performed using RevertAid reverse transcriptase (Thermo Fisher Scientific Baltics UAB, Vilnius, Lithuania). Extraction of DNA followed the protocol of Doyle and Doyle (Doyle and Doyle, 1990). For the amplification of *StFATA* from DNA and cDNA, the primers StFATA-cDNA_for and StFATA-cDNA_rev (**Table 1**) were used applying the following protocol: denaturation of 2 minutes at 98 °C, followed by a 35 times cycle of denaturation (10 sec, 98 °C), annealing (10 sec, 62 °C) and polymerisation (1 min/kb, 72 °C). Amplification was finished with a final polymerisation for 2 minutes at 72 °C. Q5 High-Fidelity DNA Polymerase (NEB, Ipswich, USA) was used for all amplifications. PCR products were cloned into pGEMT (Promega, Walldorf, Germany) and sequenced using whole plasmid sequencing by Eurofins Genomics (Ebersberg, Germany). BLAST was used to identify homologous sequences in NCBI and Spud DB (Hamilton et al., 2025).

**Table 1:** Primers used in this study.

Use	Primer name	Sequence (5ʼ-3ʼ)	T_A_ in °C	Amplicon length
A	FATA2b Forward	GGTGGAGGCAATTGATCTAGG	61	990 bp
A	FATA2b Reverse	TCTTCCATCAATCGACCAAAC	60	
A			60	672 bp
A	LBb1.3	ATTTTGCCGATTTCGGAAC	54	
B	StFATA-5UTR_for	TGCGCTTATCTCTCTCGCCTCA	59.7	5080 bp
B	StFATA-3UTR_rev	AAAGGGAGCTCCTCCACCGAGT	61.1	
B	StFATA-cDNA_for	TGTCGCCATTGGAGATCATA	52.8	999 bp
B	StFATA-cDNA_rev	TGCAAGAAGCTCTTGTTTACAT	52.7	

A) to identify *A. thaliana FATA2* mutants (SALK_111046C). Primer combination FATA2b Forward/FATA2b Reverse was used to identify wild type Arabidopsis plants without T-DNA insertion, while primer combination FATA2b Reverse/LBb1.3 detects mutant plants; B) cloning of FATA from genomic DNA or cDNA; primers names, sequences, annealing temperature (T_A_) and expected lengths of the amplified PCR fragments are given.

### 2.7 Haplotyping genes in tetraploid potatoes

To create haplotype resolved sequences for the location of *StFATA* whole genome sequences of Albatros (SRR14400529) and Désirée (SRR37799278) were analysed with the program Ranbow (Version 2.0) (Moeinzadeh et al., 2020). Ranbow was specifically developed to assemble haplotypes for heterozygous polyploid genomes. The parameters used for the execution of Ranbow were “ranbow.py hap -mode index” and “ranbow.py hap -mode hap”. Furthermore, Ranbow also uses a configuration file with more specific parameters. The parameters in the configuration file were set to “-ploidy 4” and “-noProcessors 1”. To ensure that Ranbow only processes the targeted sequence locations and to prevent formatting issues, freebayes (Version v0.9.21) was applied for variant calling of the candidate gene region (Garrison and Marth, 2012). Due to *S. tuberosum* being tetraploid, the –p parameter was set to four, as well as the –F parameter to 0.05 for a frequency-based input threshold. The –r parameter was used to restrict the variant calling to the candidate gene region “-r ST4.03ch06:59100000.59300000”. The output fasta-files from Ranbow were remapped to the *S. tuberosum* Group Phureja DM 1-3–516 R44 genome using bowtie2 (Version v2.4.4) and then sorted with samtools (Version 1.14) (Langmead and Salzberg, 2012; Danecek et al., 2021).

To verify the results from Ranbow, cDNA and genomic DNA of *StFATA2* of the cultivars Albatros and Désirée were amplified, cloned and sequenced using whole plasmid sequencing to obtain cultivar specific haplotype sequences. For further analyses, the sequences were also compared to the published 10 haplotype phased European potato genomes in Sun et al. (2025) and the haplotype genome assembly of Désirée (Godec et al., 2025).

### 2.8 Calculation of the dosage effect

The dosage effect was calculated using the exact Fisher test. DRYM ranked cultivars with two alternative alleles for the SNP at 4,439 bp in *StFATA* were grouped as well as cultivars with less than two and more than two alternative alleles. To visualize the dosage effect, boxplots were created in R using the function boxplot().

### 2.9 Protein structure analyses

Prediction of protein 3D structure was performed with the program SWISS-MODEL Version 2025_3 (Waterhouse et al., 2018) using *StFATA* sequences of the cultivars Albatros and Désirée. Visualization and calculation of cavities was done with UCSF ChimeraX Version 1.10.1 (Meng et al., 2023). A probe radius of 2.45 Å was used for the oleoyl fatty acid chain. The protein structure of each haplotype derived from Albatros and Désirée was also compared to the published haplotypes from Sun et al. (2025).

### 2.10 Characterization of *fata2* mutants

DNA was extracted from *A. thaliana* using CTAB (Edwards et al., 1991). For the verification of mutants and wild type plants PCR amplification were performed using two primer pairs (**Table 1**). The primers FATA2b Forward and FATA2b Reverse bind to the intact *FATA2* gene (AT4G13050.1), while LBb1.3 binds to the T-DNA sequence and was combined with FATA2b Reverse to identify the mutants. The PCR consisted of an initial denaturation step of 2 min at 98 °C, followed by 35 cycles of denaturation 10 sec at 98 °C, annealing 10 sec at 58 °C, extension 30 sec/kb at 72 °C and a final extension step 2 min at 72 °C. All sequenced PCR products were amplified with Q5 High-Fidelity DNA Polymerase (NEB, Ipswich, USA). The PCR products were cloned into pGEMT Easy vector according to the manufacturer’s instruction (Promega, Walldorf, Germany), which was then used to transform *Escherichia coli* DH5α. Plasmids were isolated using the GeneJet Plasmid Miniprep kit (Thermo Fisher Scientific Baltics UAB, Vilnius, Lithuania) and sequenced using the primers M13for and M13rev by Eurofins Genomics (Ebersberg, Germany) to verify the location of the T-DNA insertion and the sequence of the wild type gene.

### 2.11 Drought and recovery experiments in Arabidopsis

After stratification, plants were grown in a climate chamber under long day conditions with 16 hours daylight/8 hours night cycle at a light intensity of 100-150 µmol·m^-2^·s^-1^ at 20 °C/22 °C. Six-week-old Arabidopsis *fata2* knockout mutants and control wild types Colombia-0 were used for drought and recovery experiments. Before the drought period, plants (n=30 for each treatment) were watered every other day. To generate drought stress watering was stopped for seven days for half of the plants. Watering was continued for all plants after the drought treatment for the recovery experiments. Recovery was evaluated after one week.

### 2.12 Cuticle permeability measured by chlorophyll leaching

Chlorophyll leaching analyses from leaves of five-week-old well-irrigated plants were performed to test the cuticle permeability as described by Lü et al. (2011). Leaves of Arabidopsis wild type *Col-0* and *FATA2*-mutants were harvested and weighed (Minimum weight ≥ 100 mg). Then the leaves were incubated in 10 ml 80% ethanol at room temperature, protected by light with tin foil. While continuously shaking (45 rpm) samples (400 µl) were taken at intervals of 10, 20, 30, 40, 50, 60, 120 and 180 minutes to detect the leaked-out chlorophyll concentration in the solution. Samples were returned after the measurements. Chlorophyll absorbance was determined at 647 nm and 664 nm. The following equation was used to calculate chlorophyll/g leaf for the different time points (Pérez-Patricio et al., 2018).


$$C=[19.536\times (A_{647\;nm})+7.936\times \left(A_{664\;nm}\right)]g^{-1}$$

Finally, the leaves were left in 80% ethanol for two days, to determine the total chlorophyll content of the leaves. Subsequently, t-tests were performed (p ≤ 0.05).

## 3 Results

### 3.1 Identification and characterization of acyl-ACP thioesterases in potato

**Figure 1:**
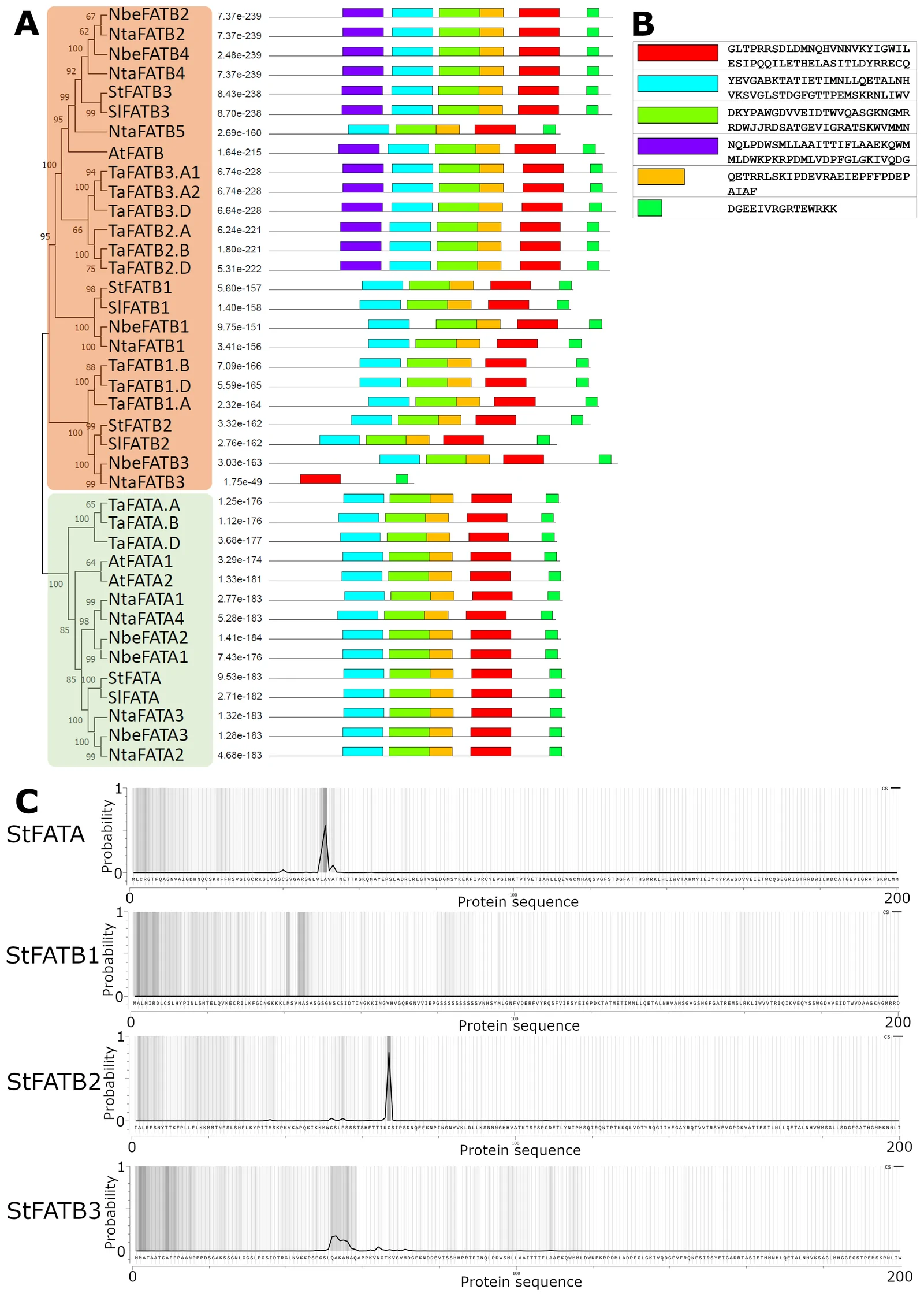
**(A)** Phylogenetic tree of *FATAs* and *FATBs* in potato, tomato, Arabidopsis, *N. tabacum, N. benthamiana* and wheat, with added motif locations, identified with Multiple Em for Motif Elicitation (MEME Suite) using the corresponding amino acid sequences. Mega12 and Maximum-likelihood method were used for the calculation of the phylogenetic tree. **(B)** Motif sequences. **(C)** Transfer peptides identified with TargetP-2.0 for *StFATA, StFATB1, StFATB2* and *StFATB3*.

The presence of acyl-ACP thioesterases were studied in *S. tuberosum* DM phureja, *S. lycopersicum*, *A. thaliana*, *N. tabacum*, *N. benthamiana* and *T. aestivum* (**Figure 1**). Applying HMMER and blastp revealed that four acyl-ACP thioesterases are present in *S. tuberosum* Group Phureja DM 1-3–516 R44 (Pham et al., 2020), as well as four in *S. lycopersicum* (Bahadir et al., 2024). In contrast, *A. thaliana* has only three acyl-ACP thioesterases, two FATA - AtFATA1 and AtFATA2 - and one AtFATB1. The only copy of *FATA* (Soltu.DM.06G033680.1) present in potato shows the highest homology to *AtFATA2* (79.2% identity) and is located on chromosome 6. The three copies of FATB (Soltu.DM.03G020290.1, Soltu.DM.05G004350.1, and Soltu.DM.12G024780.1) are distributed over chromosome 3, 5 and 12, respectively (**Table 2**). This is identical to *Solanum lycopersicum* (SlFATA, SlFATB1, SlFATB2 and SlFATB3). **Table 3** compares the gene ontology of acyl-ACP thioesterases in *S. tuberosum* and *A. thaliana*.

**Table 2:** General information about FATA and FATB genes and encoded protein in *S. tuberosum*.

FATA gene name	Gene ID	Chr	Start	End	Strand	No. of aa	MW in Dalton	pI value	No of Exons	No. of introns
StFATA1	Soltu.DM.06G033680.1	6	57933541	57938992	+	369	41670.29	5.98	7	6
StFATB1	Soltu.DM.05G004350.1	5	3751863	3748667	–	400	45881.47	7.7	6	5
StFATB2	Soltu.DM.03G020290.1	3	45249892	45252283	+	374	42094.95	8.64	6	5
StFATB3	Soltu.DM.12G024780.1	12	54749561	54744163	–	420	46574.05	6.21	6	5

Gene name, ID, chromosome, start and end on the chromosome in bp, direction of the genes on the chromosomes and number of exons and introns are shown. In addition, number of amino acids, molecular weight and pI value of the protein product are listed.

**Table 3:** Gene ontology of FATA and FATB genes in *S. tuberosum*.

Gene name	GO annotation			Ortholog in Arabidopsis	
	Molecular function	Biological process	Cellular localization	Gene name	Gene ID
StFATA1	binding, transferase activity, hydrolase activity	lipid metabolic process, cellular process, biosynthetic process	chloroplast	AtFATA1	AT3G25110.1
				AtFATA2	AT4G13050.1
StFATB1	binding, transferase activity, hydrolase activity	lipid metabolic process, cellular process, biosynthetic process	chloroplast	AtFATB	AT1G08510.1
StFATB2	binding, transferase activity, hydrolase activity	lipid metabolic process, cellular process, biosynthetic process	plastid		
StFATB3	binding, transferase activity, hydrolase activity	lipid metabolic process, cellular process, biosynthetic process	plastid		

The table shows GO terms for molecular function and biological processes of *StFATA1, StFATB1, StFATB2* and *StFATB3*, subcellular localizations and orthologs in *A. thaliana*.

While *N. tabacum* has four FATAs (*NtaFATA1, NtaFATA2, NtaFATA3, NtaFATA4*), *N. benthamiana* has only three (*NbeFATA1, NbeFATA2, NbeFATA3*). However, *N. tabacum* has five copies of FATB (*NtaFATB1, NtaFATB2, NtaFATB3, NtaFATB4, NtaFATB5*), and *N. benthamiana* only four (*NbeFATB1, NbeFATB2, NbeFATB3, NbeFATB4*). *T. aestivum* as an allohexaploid carries twelve acyl-ACP thioesterases, three FATA (*TaFATA.A, TaFATA.B, TaFATA.D*) and nine FATB genes (*TaFATB1.A, TaFATB1.B, TaFATB1.D, TaFATB2.A, TaFATB2.B, TaFATB2.D, TaFATB3.A1*, *TaFATB3.A2* and *TaFATB3.D*). For wheat, the acyl-ACP thioesterases are evenly distributed over all subgenomes with the exception of *TaFATB3*, which is present twice on subgenome A, once on subgenome D but not on subgenome B. The analysis of phylogeny confirmed, that the acyl-ACP thioesterases in *A. thaliana*, *S. tuberosum*, *S. lycopersicum*, *N. tabacum*, *N. benthamiana* and *T. aestivum* can be categorized in two groups: FATA and FATB (**Figure 1A**). Out of all 39 acyl-ACP thioesterases 14 belong to the group FATA and 25 to FATB.

### 3.2 FATA gene structure and conserved motifs

Analysing the amino acid sequences of the investigated 39 acyl-ACP thioesterases with the Multiple EM for Motif Elicitation (MEME) suite version 5.5.7 (Bailey and Elkan, 1994) showed five motifs present in 38 proteins, which might have core functions (**Figure 1B**). Besides, one additional motif was present in 13 of the acyl-ACP thioesterases (StFATB3, SlFATB, TaAFATB2, TaBFATB2, TaDFATB2, TaAFATB3, TaBFATB3, TaDFATB3, NtaFATB2, NtaFATB4, NtaFATB5, NbeFATB2, NbeFATB4) and one carried only one of the additional domains (NtaFATB5). All acyl-ACP thioesterases, except NtaFATB3, shared a common conserved domain PLN02370 (hotdog domain), which is crucial for the function. With only two motifs NtaFATB3 is an exception. This might suggest a false protein annotation or major deletions in the 5’ end of the gene. The motifs identified by MEME ranged from 15 to 50 amino acids. Using NCBIs CDD, the red and green motif were identified as part of the PLN02370 and hotdog domain. Orange, mint and teal are also part of the PLN02370 but not the hotdog domain. And the purple motif was identified as the acyl-thio N domain. Using TargetP 2.0, chloroplast transfer peptide cleavage sites were located at 51–52 and 53–54 amino acids in *StFATA* and *StFATB3*, respectively, with a probability of 73% and 92% (**Figure 1C**). For *StFATB2* a transfer peptide cleavage site could not be identified at first. After adding 90 nucleotides to the 5’ terminus of the annotated *StFATB2* a chloroplast transfer peptide cleavage site was identified at 67–68 amino acids with a probability of 57%. Even after adding 5’nucleotide sequences to *StFATB1*, no cleavage site was identified.

StFATA has an amino acid (aa) length of 369, whereas the larger StFATBs range in their amino acid length from 374 to 420 amino acids and in average of 398 aa (**Table 2**). The molecular weight of StFATA is 41670.29 Da and for the StFATBs it ranges from 42094.95 to 46574.05 Da, with an average of 44850.16 Da. The isoelectric point of StFATA is the lowest with 5.98, whereas for the StFATBs it ranges from 6.21 to 8.64, with an average of 7.52.

### 3.3 FATA cis regulatory elements

The 5’-upstream region (2,000 bp) of the genes *StFATA*, *StFATB1*, *StFATB2* and *StFATB3* were analysed for drought related motifs and compared to each other (**Supplementary Tables 1**–**6**). In total, 2134 cis-acting elements were identified, of which 482, 451, 470 and 731 motifs were present in the 2,000 bp upstream of *StFATA, StFATB1, StFATB2* and *StFATB3*, respectively. The ten most abundant cis regulatory elements (CRE) for the promoter region of the four genes are highly similar. Five motifs, CACTFTPPCA1, CAATBOX1, DOFCOREZM, GT1CONSENSUS, ARR1AT, are in the top ten of all *StFAT* genes (**Supplementary Table 7**). For *StFATA*, 91 unique CREs were identified, with 18.68% related to drought stress. While in the ten most abundant list for *StFATA* only the motif MYCCONSENSUSAT is unique. ROOTMOTIFTAPOX1, an element of root specificity, is highly represented in the ten most abundant CREs of the three FATB genes, but also present with about half the frequency in FATA of Désirée. The comparison of the promoter regions of the cultivars Albatros and Désirée shows small differences in the number of cis regulatory elements, but Albatros has one additional regulatory element called SEF4MOTIFGM7S in the top ten that is related to drought stress.

### 3.4 Differential expression of *FAT* genes in potato under drought stress

In the drought-tolerant cultivar Lady Rosetta, the expression of *StFATA* (PGSC0003DMG400020163/Soltu.DM.06G033680.1) in leaves increased significantly under drought stress in the field trials after 56 days of drought with a log_2_FC of 1.13 and an adjusted p-value of 0.03 (**Table 4**). No differences were reported for the *FATB* genes (Aliche et al., 2021).

**Table 4:** Differential expression of StFATA, StFATB1, StFATB2 and StFATB3 in leaves after days of drought (DOD) treatment.

Cultivar	Gene	Location	Treatment	Mean [FPKM]	log_2_FC	Adjusted p-value
Lady Rosetta	FATA	Field	56 DOD	641.58	1.12	0.03
Désirée	FATA	Field	60 DOD	10.67	0.28	0.07
Désirée	FATA	Greenhouse	60 DOD	8.89	0.33	0.11
Désirée	FATB1	Field	60 DOD	0.19	1.74	0.28
Désirée	FATB1	Greenhouse	60 DOD	0.093	0.48	0.73
Désirée	FATB2	Field	60 DOD	0	0	1
Désirée	FATB2	Greenhouse	60 DOD	0	0	1
Désirée	FATB3	Field	60 DOD	86.72	0.09	0.71
Désirée	FATB3	Greenhouse	60 DOD	97.11	0.02	0.91

In the cultivar Désirée, *StFATA* showed no differences in the expression level in leaves between drought and control on the field or in the greenhouse. The expression of *StFATB1* was increased with a log_2_FC of 1.74 (field), but was not significantly different between the treatments. *StFATB2* was not expressed at all and *StFATB3* did not change under drought stress.

### 3.5 *StFATA* haplotypes in Albatros and Désirée

**Figure 2:**
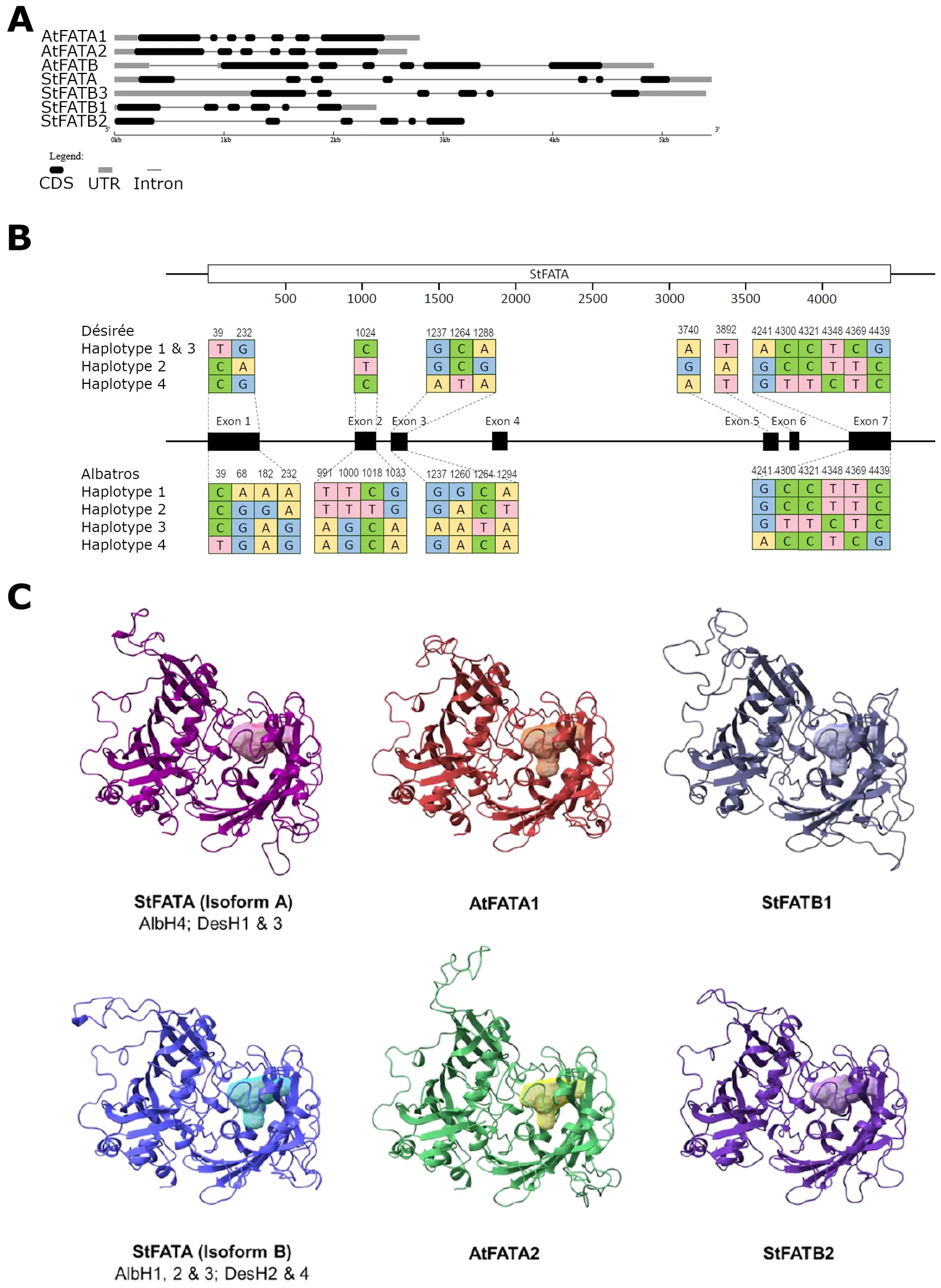
**(A)** Exon-intron overview of genes *AtFATA1, AtFATA2, ATFATB, StFATA, StFATB1, StFATB2* and *StFATB3*. Exons are depicted in black and UTR regions in grey. **(B)** Haplotype overview of *StFATA* in potato cultivars Albatros and Désirée, showing the polymorphisms in exons of all haplotypes. **(C)** Protein structure predictions of *FATA* and *FATB* in potato and Arabidopsis, showing the differences in the binding pocket of predicted haplotype isoforms A and B in the potato cultivars Albatros and Désirée. Structure visualization was done with SWISS-MODEL and binding pocket calculations with ChimeraX.

The exon-intron structures between the acyl-ACP thioesterase genes in Arabidopsis and potato show considerable variations (**Figure 2A**). For example, *StFATA* spans 4857 bp and consists of 7 exons, whereas *AtFATA2* is 1617 bp long and has only 6 exons. To create haplotype resolved sequences of the *FATA* gene in potato, *FATA* from the cultivars Désirée and Albatros were amplified from cDNA and genomic DNA, cloned and sequenced using whole plasmid sequencing. Four haplotypes were identified for Albatros and three for Désirée (**Figure 2B**). These results were supported with haplotype structures created with ranbow based on whole genome sequencing data (Schilling et al., 2024). In addition, the identified haplotypes were verified by comparing the sequences with the 10 haplotype resolved genomes from the potato pan genome (Sun et al., 2025) and the haplotype resolved Désirée genome assembly (Godec et al., 2025). All sequences had a deletion of 386 bp in comparison to the reference sequence Group Phureja DM 1-3–516 R44 (Pham et al., 2020) in the intron between exon 1 and exon 2. The different haplotypes in Albatros and Désirée differ only by SNPs except haplotype 1 of Albatros, which is characterized by an additional insertion of 31 bp between exon 4 and 5. Albatros haplotype 4 and Désirée haplotypes 1 and 3 are identical (**Figure 2B**). These haplotypes share four unique SNPs at position C39T, G4241A, T4369C, C4439G. In addition, Albatros haplotype 3 and Désirée haplotype 4 are identical. All Désirée haplotypes and Albatros haplotypes 1, 3 and 4 were also present in the potato pan genome by Sun et al. (2025), while Albatros haplotype 2 is unique and has not been described before (**Table 5**). Haplotype 2 of Albatros is characterized by polymorphisms in exon 1, 2 and 3 at 182 bp (A > G), 1018 bp (C > T) and 1294 bp (A > T), respectively. Thereby haplotype 2 in Albatros resembles in these positions the reference genome sequence. No other haplotype of the cultivars Albatros and Désirée shows these polymorphisms (**Table 5**). The allelic distribution for specific SNPs from SeqSNP and the DRYM ranking of 34 cultivars (Schilling et al., 2024) allowed the calculation of dosage effects on the drought tolerance. The SNP C4439G in *StFATA*, which is present in haplotype 4 of Albatros and haplotypes 1 and 3 of Désirée, showed a dosage effect for drought tolerance in cultivars with the haplotype combination C/C/G/G. (**Supplementary Table S8**; **Supplementary Figure 1**).

**Table 5:** SNP position and type of polymorphisms in all four Désirée and Albatros haplotypes.

Cultivar	Ref.			Désirée				Albatros				Isoform	
Pos. (DNA)	Pos. (Gene)	Pos (Prot)	Type of Polymorphism	H1	H2	H3	H4	H1	H2	H3	H4	A	B
C39T	39	Ala13Ala	Synonymous	T	C	T	C	C	C	C	T		
G68A	68	Arg23Lys	Missense	–	–	–	–	A	G	G	G		
**A182G**	182	Lys61Arg	Missense	–	–	–	–	A	**G**	A	A		
G232A	232	Val78Met	Missense	G	A	G	G	A	A	G	G		
A991T	1380	Gly123Gly	Synonymous	–	–	–	–	T	T	A	A		
G1000T	1389	Thr126Thr	Synonymous	–	–	–	–	T	T	G	G		
**C1018T**	1407	Thr132Thr	Synonymous	–	–	–	–	C	**T**	C	C		
C1024T	1413	Ser134Ser	Synonymous	C	T	C	C	–	–	–	–		
G1033A	1422	Lys137Lys	Synonymous	–	–	–	–	G	G	A	A		
G1237A	1626	Gly174Gly	Synonymous	G	G	G	A	G	G	A	G		
A1260G	1649	Lys182Arg	Missense	–	–	–	–	G	A	A	A		
C1264T	1653	Asp183Asp	Synonymous	C	C	C	T	C	C	T	C		
A1288G	1677	Gly191Gly	Synonymous	A	G	A	A	–	–	–	–		
**A1294T**	1683	Ala193Ala	Synonymous	–	–	–	–	A	**T**	A	A		
G3740A	4085	Ser251Ser	Synonymous	A	G	A	A	–	–	–	–		
A3892T	4239	Val277Val	Synonymous	T	A	T	T	–	–	–	–		
G4241A	4604	Asp287Asn	Missense	A	G	A	G	G	G	G	A	A (Asn)	G (Asp)
C4300T	4663	Asp306Asp	Synonymous	C	C	C	T	C	C	T	C		
C4321T	4684	Ser313Ser	Synonymous	C	C	C	T	C	C	T	C		
T4348C	4711	Ala322Ala	Synonymous	T	T	T	C	T	T	C	T		
T4369C	4732	Asn329Asn	Synonymous	C	T	C	T	T	T	T	C		
C4439G	4802	Leu353Val	Missense	G	C	G	C	C	C	C	G	G (Val)	C (Leu)

The first column shows the genic position (Pos.) to the sequenced DNA of Désirée and Albatros. The second column shows the position according to the gene annotation of the reference genome DM v6.1 (Ref.). Nucleotide exchanges specific for the unique haplotype 2 in Albatros are shown in bold. – denotes nucleotides not relevant for this haplotype.

On protein level, a total of six different amino acids were observed in the four haplotypes in the cultivar Albatros. The most severe changes in amino acid sequences were present in haplotype 4 with Asp287Asn and Leu353Val, which differs from all other Albatros haplotypes. However, these changes are also present in haplotype 1 and 3 of Désirée. Furthermore, haplotype 2 from Désirée and haplotypes 1 and 2 from Albatros share an amino acid exchange Val78Met. All other missense amino acid exchanges Arg23Lys, Lys61Arg, and Lys182Arg are conservative. In addition, 16 synonymous SNPs are present in the identified haplotypes. Comparison of the amino acid sequence homologies of the identified haplotypes of Albatros and Désirée shows distinct features of StFATA with regard to StFATB1, StFATB2 and StFATB3 (**Supplementary Figure 2**).

### 3.6 *StFATA* protein structures

Hypothetical secondary protein structures were predicted with ChimeraX using the haplotype sequences. Albatros haplotype 4 and Désirée haplotype 1 and 3 could be distinguished in their hypothetical secondary structure and calculated cavities from all other haplotypes (**Figure 2C**). Comparing the structure of StFATA to the related StFATB1, StFATB2, and StFATB3 shows that the predicted cavities from Albatros haplotype 4 and Désirée haplotype 1 and 3 of StFATA (hypothetical isoform A) are similar to StFATB2, while all other haplotypes of StFATA (Albatros 1, 2 and 3 as well as Désirée 2 and 4) have predicted cavity structure similar to StFATB1. All hypothetical binding pockets included the conserved catalytic amino acids Asp263, Asn265, His267 and Glu301, which surround the pocket opening (Aznar-Moreno et al., 2018). The polymorphism Asp287Asn is located at the opening of the binding pocket in the hypothetical isoform A (**Supplementary Figure 3**). The lower part of the predicted extended cavity, visible in the hypothetical isoform B, is located opposite the catalytically active opening of the pocket and is approximately 14 Å away from the top. Due to the absence of the predicted extended cavity region in the hypothetical isoform A, the distance between the top of the pocket opening and its lowest point would only be about 10 Å apart, which would be too small for C18:1 fatty acid molecule.

### 3.7 Drought and recovery in Arabidopsis *fata2* knockout mutants

**Figure 3:**
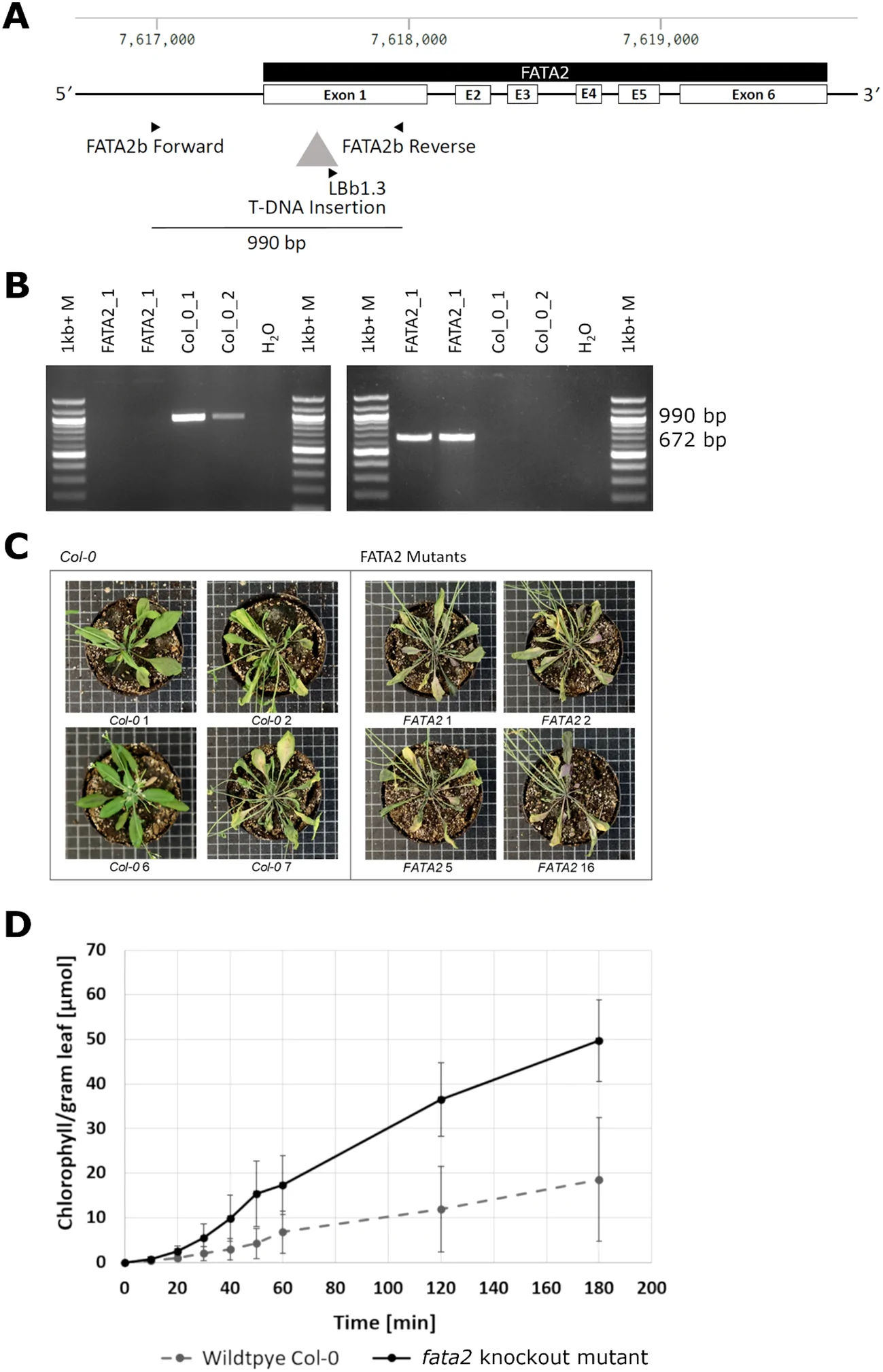
**(A)** Scheme of the location of the T-DNA mutation in *AtFATA2* (AT3G25110.1) in *A. thaliana* (SALK_111046C) including the location of the applied primers. Exons are indicated as white boxes, primers as black arrows and the T-DNA insertion as a grey arrow. **(B)** Screening for the presence of the T-DNA mutation in T1 generation of SALK_111046C with primer combination FATA2b Reverse and LBb1.3 (right side) and the intact gene *AtFATA2* with the primer combination FATA2b Forward and FATA2b Reverse (left side). The primers FATA2b Forward and FATA2b Reverse amplify a 990 bp long fragment. The primers FATA2b Reverse and LBb1.3 produce an amplicon of 672 bp, with primer LBb1.3 binding to the T-DNA. **(C)** Drought stress experiment of Arabidopsis Col-0 wildtype and *fata2* knockout mutants after seven days without irrigation. **(D)** Comparison of the chlorophyll leaching per gram leaf mass of Arabidopsis Col-0 wildtype plants (grey) and *fata2* knockout mutants (black). Time in minutes is shown on the x-axis and μmol chlorophyll per gram leaf mass on the y-axis.

A knockout *fata2* mutant (SALK_111046C) was used to study the effect of the T-DNA mutation on drought tolerance in Arabidopsis. Homozygosity of the mutants as well as the absence of the wild type gene were confirmed by gene-specific PCR (**Figures 3A, B**).

Six-week-old Arabidopsis *fata2* knockout mutants (n=30) and control wild types (n=30) were used for the drought experiments. Watering was stopped for seven days. A total of 55.6% wild type plants survived seven days of drought, whereas only 26.2% of the *fata2* knockout mutants were still alive (**Figure 3C**). For the recovery experiments, all plants were watered again after seven days, but no differences in the regeneration were observed after another week.

### 3.8 Cuticle permeability analysis

To determine the influence of FATA2 on the permeability of the cuticle, chlorophyll leaching over a period of time (10, 20, 40, 60, 120 and 180 min) was measured. Using three medium-sized leaves per plant, significant differences in leaked chlorophyll per gram of leaf were detectable for all measurement points (**Figure 3D**) except at the beginning, after 10 minutes, and at the end, after 48 hours (**Table 6**). All other measurement points were significantly different between *fata2* knockout mutant and control plants with p-values < 0.05 (**Table 6**).

**Table 6:** Two-sample t-test comparing leaked chlorophyll concentration from wildtype and FATA2 mutants after specific time points.

Time [min]	Col-0 mean ± Sdev (n=3)	*fata2*-mutant mean ± Sdev (n=3)	p-value
10	0.46 ± 0.48	0.70 ± 0.28	0.24
20	1.03 ± 0.64	2.51 ± 1.18	0.0066
30	2 ± 1.57	5.45 ± 3.16	0.0138
40	2.94 ± 2.44	9.86 ± 5.17	0.0035
50	4.25 ± 3.34	15.42 ± 7.33	0.0012
60	6.78 ± 4.66	17.35 ± 6.60	0.0019
120	11.96 ± 9.60	36.49 ± 8.24	0.00005
180	18.59 ± 13.86	49.74 ± 9.12	0.00007
After 48 hours	70.95 ± 9.68	77.11 ± 6.75	0.159

After 60 minutes, the average loss of chlorophyll for the *fata2* knockout mutants reached a value of 17.35 µmol chlorophyll per gram leaf mass (**Figure 3D**). Whereas the wild type control lost only 6.78 µmol chlorophyll per gram leaf mass in the same time. This represents a 39% higher loss of chlorophyll for the *fata2* knockout mutant. At 180 minutes, the wild type still showed a 37% lower loss of chlorophyll per gram leaf mass than the knockout mutant. After 48 hours, the loss of chlorophyll for the wild type and the knockout mutant was at 70.95 and 77.1 µmol chlorophyll per gram leaf mass, respectively. This showed that the total chlorophyll content per leaf was not significantly different (**Figure 3D**), but the cuticle permeability in the *fata2* knockout mutants was severely impaired compared to the wild type. However, to test the hypothesis that the increased cuticle permeability was due to changes in the cuticle composition and/or cuticle structure, additional chemical analyses of the cutin and wax contents of the cuticle need to be performed as well as structural analyses of the cuticle.

## 4 Discussion

### 4.1 Variation of *FATA* and *FATB*

Based on the substrate-specificity and the amino acid composition, acyl-ACP thioesterases have been divided into two distinct groups in plants (Voelker et al., 1997; Salas and Ohlrogge, 2002). In potato, the four acyl-ACP thioesterase genes also group in these two phylogenetically distinct subfamilies: *FATA* (one member) and *FATB* (three members). Most plant species e.g. like *A. thaliana* (Jones et al., 1995), or even walnut (Wang et al., 2024c) have at least two *FATA* genes, but *S. tuberosum* and the closely related *S. lycopersicum* show only a single copy of *FATA* (Bahadir et al., 2024). The close relationship between potato and tomato can also be seen in the distribution of *FATA* and *FATB* genes on the chromosomes and the genetic structure of the corresponding genes, which are almost identical. The higher number of acyl-ACP thioesterases in *N. benthamiana* and *N. tabacum* can be explained by their allotetraploid origin (Schiavinato et al., 2020; Zan et al., 2025), as for the allohexaploid wheat (International Wheat Genome Sequencing Consortium (IWGSC, 2018). Analyses of cis regulatory elements showed that motifs found in drought stress related genes are common in the promoter region of *StFATA* and *StFATBs*. The cis regulatory element MYCCONSENSUSAT in *StFATA* is directly tied to drought stress in plants, supporting its possible role in the drought stress response (Baldoni et al., 2015; Negroni et al., 2024).

Additionally, RNAseq analyses showed significantly increased expression rates in leaves for *StFATA* under water deficit in the cultivar Lady Rosetta in field trials, but not in the cultivar Désirée. For the *StFATB* genes, no significant expression differences in leaves were observed in both cultivars. Differences in the expression levels of *StFATA* in leaves might be due to differences in the application of the drought stress, or might reflect different mechanisms to cope with drought, in which StFATA might play a role.

### 4.2 *StFATA*-haplotypes

Deriving haplotypes from autotetraploid potato genome sequences proves challenging and only recently haplotype phased potato genomes have been published (Godec et al., 2025; Sun et al., 2025). To understand the role of FATA in drought tolerance of potato it was essential to derive haplotypes for the two investigated cultivars that were also well characterized with regard to their drought tolerance (Schilling et al., 2024). Analyses of cDNA and genomic sequences obtained by whole plasmid sequencing allowed the definition of *StFATA* haplotypes in the cultivars Albatros and Désirée. All SNPs were compared and verified by whole genome sequencing (Schilling et al., 2024). The BSA-Seq data analyses combined with SeqSNP analyses in an association panel of 34 potato cultivars had allowed the identification of drought-stress-associated genes such as *StFATA*, and also the assignment of individual SNPs to the corresponding haplotypes in the drought-sensitive and drought-tolerant bulks (Schilling et al., 2024). To investigate the hypothesis that the identified SNPs might have functional relevance for *StFATA*, SNP-based haplotypes were defined, their amino acid sequences compared, and the hypothetical protein structures were predicted by ChimeraX. One SNP (C>G) proved to be significantly associated with drought tolerance, corresponding in the DNA data collected in this study to the polymorphism at position 4439 bp. This suggests that this base exchange, which leads to a missense mutation (Leu353Val) and the additional SNP (A>G) (Asp287Asn) at position 4241 bp result in the formation of the StFATA isoform A, which leads to a crucial change in the predicted morphology of the resulting proteins. Especially the polymorphism Asp287Asn is located directly at the surface of the predicted binding pocket. Both polymorphisms can alter the predicted binding pockets of StFATA, thereby probably shifting the substrate specificity towards shorter fatty acid ACPs. The predicted isoform A of haplotypes AlbH4 and DesH1 & 3, in which the lower part of the cavity is absent, may completely prevent oleoyl binding. The oleoyl (18:1 cis-Δ9) chain possesses a double bond between C9 and C10, resulting in a locally inflexible bond at this position with an angle of approximately 30°. Therefore, FATA pockets must be designed to provide sufficient space and compatibility for the hydrocarbon chain of the monounsaturated oleoyl. During binding, the fatty acid chain attached to the phosphopantetheinyl arm of ACP initially unfolds out of the ACP protein, making it accessible to the enzyme FATA (Johnson et al., 2014). Subsequently, only the hydrocarbon chain of the fatty acid binds to the binding pocket, with the carbonyl group remaining at the opening near the catalytic amino acids, where it is available for the hydrolytic cleavage of the thioester bond (Feng et al., 2017). The larger cavity of predicted isoform B might allow the hydrolysis of oleoyl-ACP. For the corresponding cavities in the enzymes AtFATA1 and AtFATA2 from *A. thaliana*, substrate preference for oleoyl-ACP have already been shown by enzyme assays (Salas and Ohlrogge, 2002; Aznar-Moreno et al., 2018). The resemblance to the active pockets of isoform B in potato, might suggest that proteins of this type might as well be capable of binding and catalysing oleoyl-ACP. This hypothesis of different substrate specificities for the two isoforms in potato has to be confirmed by enzyme activity assays to show changes in the substrate specificity.

It can be hypothesized that an enzyme, which does not release oleoyl from the ACP in the chloroplast, potentially leads to a reduction in the available unsaturated fatty acids (C18:1) in the acyl-CoA pool of the ER membrane, where they are required to synthesize important components of the cuticle. Cutin biosynthesis, in particular, could be disrupted, thus triggering the destabilization of the barrier against non-stomatal transpiration. Whether this is true has not been investigated for FATA.

Both Albatros and Désirée (Schilling et al., 2024) represent drought tolerant cultivars, which in case of Albatros possesses one haplotype leading to the predicted StFATA isoform A and two haplotypes in Désirée. The analysis of the dosage effect indicates that two dosages of the isoform A might be ideal for drought tolerance in potato. However, whether different isoforms of FATA in potato lead to changes in the cuticle is unknown. Up to now, the cuticle of the potato leaves has not been analysed regarding the chemical composition and cuticle structure. However, this is essential for further conclusions. In which way different isoforms of FATA might have an influence on the cuticle formation needs to be further investigated in detail. This can be either done by applying genome editing, especially base-editing, to have one type of haplotype present to see the effect or to obtain a knockout of *StFATA* to analyse the loss of function in potato. In addition, overexpression of the different FATA haplotypes in potato would also give a deeper insight into the function. Another possibility would be also to compare the cuticles of cultivars Kolibri and Karlena (C/C/G/G), with cultivars having either all G/G/G/G like Saturna and Golf or cultivars with all C/C/C/C like Ramses and Euroresa from the association panel. For the latter two whole genome sequences are already available. Analyses in tetraploid potato proves challenging as seen in our study. However, a allele dosage effect has already been demonstrated for the intensity of the red skin pigment in potato by using a SNP marker associated with tuber skin colour (De Jong et al., 2003). To bypass the complex tetrasomic inheritance of potato the development of diploid inbred lines is gaining interest (Clot et al., 2024; Vexler et al., 2024).

### 4.3 FATAs influence under drought stress

Analysing *fata2* Arabidopsis knockout mutants after seven days of drought resulted in a significantly lower survival rate compared to wild type plants (26.2% versus 55.6%). The hypothesis that the acyl-ACP thioesterase A might play a role in cuticle formation and thereby drought tolerance has to be verified by analyses of the cuticle structure and chemical composition. The measurements of the chlorophyll leaching indirectly supports this, hinting towards possible cuticle defects in the Arabidopsis *fata2* knockout mutants. The chlorophyll leaching results revealed significant differences between wild type and *fata2* knockout mutant plants within the incubation period of 20 to 180 minutes, with wild type plants showing lower chlorophyll leaching than the *fata2* knockout mutants. For studies regarding the cuticular barrier based on its chemical composition, Arabidopsis mutants of the wax and cutin biosynthesis (*att1*, *bdg*, *cer5*, *cut1*, *shn1*, *shn3*, *wax2*) have been compared to the corresponding wild types (Sadler et al., 2016). Some of the genetic changes impaired the cutin and wax deposition and led to lowered barrier properties of the cuticles in leaves. However, increasing the amount of wax and cutin by transgenic approaches did not automatically result in improved barrier properties of the cuticles. Radioisotopes as ^14^C-labeled epoxiconazole were used as tracers, but also chlorophyll fluorescence was tested for measurement of the cuticle barrier properties (Sadler et al., 2016).

FATAs play a crucial role in the fatty acid biosynthesis, which is necessary for the cuticle formation (Li-Beisson et al., 2013; Joubès and Domergue, 2018). Mutants of the cuticle biosynthesis result in a weakened defence barrier between plant and environment (Lee et al., 2009; Xie et al., 2020). In our study, the lack of the FATA2 enzyme in the *fata2* knockout mutants led to a higher cuticle permeability. In addition, the mutants exhibited significantly lower drought tolerance compared to the wild type. The reduced capability of the cuticle to prevent non-stomatal transpiration might explain the lower survival rate of the *fata2* knockout mutants under drought conditions. However, this did not have an apparent effect on the recovery behaviour, when the crucial stressor was eliminated by rewatering the plants. FATA2 is not known to play a significant role in plant regeneration (Wan et al., 2025) and the effect of drought stress might have been moderated in the *fata2* mutants by the fact that other enzymes, such as AtFATB1, AtFATB2 or AtFATA1, were able to compensate for the deficiency to some degree (Bonaventure et al., 2003; Moreno-Perez et al., 2012). In tomato, genome-edited knockout mutants of *SlFATB1* and *SlFATB3* could be obtained that showed differences in the leaf anatomy (Bahadir et al., 2024). A reduction in the number of stomata and trichomes was observed in both knockout mutants. In addition, the cuticle structure was changed and showed a reduction in deposited epicuticular wax (Bahadir et al., 2024). However, no knockout mutants of *SlFATA* were obtained.

While FATAs primary function is to hydrolyze oleoyl-ACP, which can be used to form the cutin network, FATA also separates to a minor percentage the saturated fatty acids palmitoyl-ACP and stearoyl-ACP from ACP (Li-Beisson et al., 2013). So, the knockout of FATA2 could also lead to a reduced formation of the water-impermeable wax layer. Saturated C16 and C18 chains are used to build alcohols and alkanes, which serve as building blocks for cuticular waxes (Bernard and Joubés, 2003). The results of our study indicate for Arabidopsis a link between FATA2 and plant drought tolerance, in the way that the enzyme might influence the composition or structure of the cuticle, which plays an essential role for protecting the plants against water loss. Both, in the direct drought stress experiment and in the indirect permeability assays, clear differences were observed between the wild type plants and the Arabidopsis *fata2* knockout mutants. However, analyses of the cuticle composition and structure have still be performed to verify a connection.

In *N. tabacum*, it was shown that maintaining high photosynthetic rates under drought stress is the key factor to drought tolerance (Feng et al., 2023; Zahra et al., 2023). This can be achieved by strengthening cuticle and thereby reducing the non-stomatal transpiration. Priming *Sorghum bicolor* seeds with abscisic acid increased the amount of leaf wax by 37.7% and cutin by 25.6%, reducing the water loss up to 36.6% under drought (Yao et al., 2025). Both an increase in cuticular waxes or an increase in cutin is beneficial for drought tolerance (Zhao et al., 2026). However, cuticle permeability also represents a significant parameter in the trade-off between drought tolerance via less non-stomatal transpiration and CO_2_ uptake through the cuticle (Monda et al., 2021).

## 5 Conclusions

In *S. tuberosum*, only one *FATA* gene is present, but three *FATB* genes as in tomato. In the promoter region of *StFATA*, drought stress related cis-regulatory elements were identified. Under drought stress the expression of *StFATA* in leaves of the cultivar Lady Rosetta was significantly increased, whereas no differences were observed in the expression of *StFATB* genes. Haplotypes for *StFATA* were designed for the cultivars Albatros and Désirée. Predicted hypothetical protein structures based on these haplotypes identified two isoforms A and B with different morphology of the predicted substrate binding pocket, which might result in different substrate specificity. This hypothesis has to be verified by enzyme activity assays. This might be interesting as a changed substrate specificity might influence the composition and/or structure of the cuticle and thereby have an impact on drought tolerance. This requires the analyses of the cuticle composition and structure in potato in combination with drought stress experiments, which so far has not been done.

In Arabidopsis, *fata2* knockout mutants showed higher cuticle permeability compared to the wildtype plants. In addition, the survival rate of the *fata2* knockout mutants under drought was drastically reduced, even though the recovery rate did not change. The compiled results hint towards a potential role of *FATA* in drought tolerance in Arabidopsis and suggest further investigations into the role of *FATA* also in other plants like potato might be interesting.

Our study helps with the decryption of the complex processes of drought tolerance in plants, and in the future might enable the development of crops that can better withstand climate change and the resulting increase in drought periods. Reduction of non-stomatal transpiration by improved cuticle properties could be one aspect to advance drought tolerance in potato plants.

## Data Availability

The datasets presented in this study can be found in online repositories. The names of the repository/repositories and accession number(s) can be found below: https://www.ncbi.nlm.nih.gov/, SRR14400529 https://www.ncbi.nlm.nih.gov/, SRR37799278.
