# Supplementary material for: Genome-wide analysis of FATA associated with drought tolerance in tetraploid potato (Solanum tuberosum)
Source: Front Plant Sci. 2026 Aug 25;17:1917461. doi: 10.3389/fpls.2026.1917461 (PMC13550143; doi:10.3389/fpls.2026.1917461)
Supplement: Supplementary file 1 [file Supplementaryfile1.docx]

**Supplementary Figure S1: Dosage effect of the SNP at 4,439 bp in *StFATA* in the 34 DRYM ranked cultivars.** The y-axis shows the DRYM ranking value and the x-axis the allele dosage as a percentage. The haplotype combination C/C/G/G has a p-value of 0.076 with the exact Fisher test.

**Supplementary Figure S2: StFATA amino acid sequence comparison of the specific haplotypes from Albatros and Désirée in comparison to FATB1, FATB2 and FATB3 amino acid sequences.**

**Supplementary Figure S3: Missense mutation Asp287Asn at the entrance of the binding pocket.**
